# Prion protein codon 129 polymorphism in mild cognitive impairment and dementia: the Rotterdam Study

**DOI:** 10.1093/braincomms/fcaa030

**Published:** 2020-03-20

**Authors:** Hata Karamujić-Čomić, Shahzad Ahmad, Thom S Lysen, Alis Heshmatollah, Gennady V Roshchupkin, Meike W Vernooij, Annemieke J M Rozemuller, Mohammad Arfan Ikram, Najaf Amin, Cornelia M van Duijn

**Affiliations:** f1 Department of Epidemiology, Erasmus MC, University Medical Center, Rotterdam, Netherlands; f2 National Prion Disease Registry, Department of Epidemiology, Erasmus MC, University Medical Center, Rotterdam, Netherlands; f3 Department of Medical Informatics, Erasmus MC, University Medical Center, Rotterdam, Netherlands; f4 Department of Radiology and Nuclear Medicine, Erasmus MC, University Medical Center, Rotterdam, Netherlands; f5 Department of Pathology, Amsterdam Neuroscience, Amsterdam UMC, Vrije Universiteit Amsterdam, Amsterdam, Netherlands; f6 Department of Pathology, UMC Utrecht, Utrecht, Netherlands; f7 Clinical Trial Service Unit and Epidemiological Studies Unit, Nuffield Department of Population Health, University of Oxford, Oxford, UK

**Keywords:** prions, mild cognitive impairment, dementia

## Abstract

Creutzfeldt–Jakob disease is a rare, fatal, neurodegenerative disease caused by the accumulation of abnormally folded prion proteins. The common polymorphism at codon 129 (methionine/valine) in the prion protein (*PRNP*) gene is the most important determinant of genetic susceptibility. Homozygotes of either allele have a higher risk of sporadic Creutzfeldt–Jakob disease. Various studies suggest that this polymorphism is also involved in other forms of dementia. We studied the association between the codon 129 polymorphism of the *PRNP* gene and mild cognitive impairment in 3605 participants from the Rotterdam Study using logistic regression analyses. Subsequently, we studied the association between this polymorphism and incident dementia, including Alzheimer’s disease, in 11 070 participants using Cox proportional hazard models. Analyses were adjusted for age and sex. We found the prevalence of mild cognitive impairment to be higher for carriers of the methionine/methionine genotype (odds ratio, 1.40; 95% confidence interval, 1.11–1.78; *P *=* *0.005) as well as for carriers of the valine/valine genotype (odds ratio, 1.37; 95% confidence interval, 0.96–1.97; *P *=* *0.08). The codon 129 polymorphism was not associated with the risk of incident dementia or Alzheimer’s disease. In conclusion, we found a statistically significant higher prevalence of mild cognitive impairment in carriers of the methionine/methionine genotype in the codon 129 polymorphism of the *PRNP* gene within this population-based study. No associations were found between the codon 129 polymorphism and dementia or Alzheimer’s disease in the general population.

## Teaser

There is increasing interest in the role of the common polymorphism at codon 129 in the prion protein gene in neurodegenerative traits. We report a role of this polymorphism in mild cognitive impairment in the general population. However, no association was found with dementia.

## Graphical Abstract

**Graphical Abstract:**
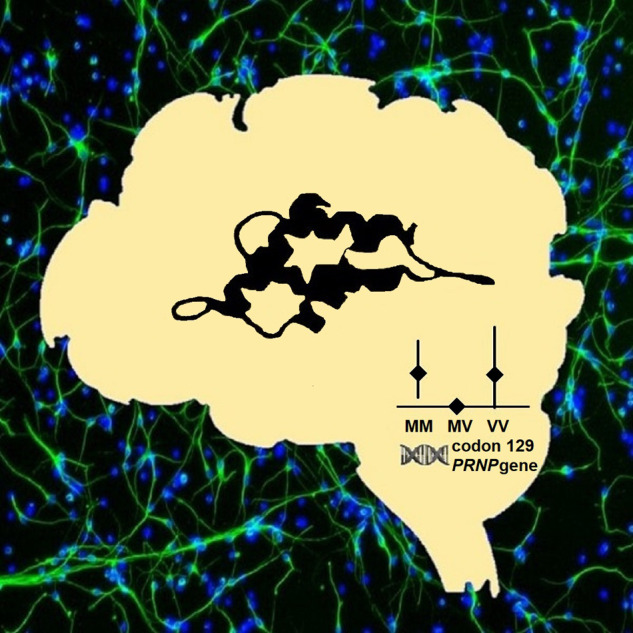

## Introduction

Creutzfeldt–Jakob disease is a rare, fatal, neurodegenerative prion disease caused by the accumulation of abnormally folded prion proteins. These proteins are the product of the prion protein (*PRNP*) gene. In prion diseases, the cellular prion protein (PrP^C^) converts into a misfolded infectious state, the pathological prion protein (PrP^Sc^) (Prusiner, 1982). Creutzfeldt–Jakob disease occurs in different forms: sporadic, genetic, iatrogenic, and variant. The sporadic form of Creutzfeldt–Jakob disease is the most common from. A major driver of the development and course of sporadic Creutzfeldt–Jakob disease is a genetic variant at codon 129 (methionine/valine) of the *PRNP* gene (Parchi *et al.*, 2012). Persons homozygous for either the methionine or valine allele are at increased risk of sporadic Creutzfeldt–Jakob disease (Alperovitch *et al.*, 1999; Parchi *et al.*, 2009). It has been speculated that homozygosity for either allele results in the synthesis of identical proteins, enabling the propagation and aggregation of *PRNP* in the brain, thus influencing both the risk and progression of sporadic Creutzfeldt–Jakob disease (Alperovitch *et al.*, 1999; Parchi *et al.*, 2009). Clinically, sporadic Creutzfeldt–Jakob disease is characterized by rapidly progressive dementia, and ultimately death on average 6 months after diagnosis. The variant form of Creutzfeldt–Jakob disease is the zoonotic form of the disease, which is transmitted through meat and other food products contaminated with material from cattle with bovine spongiform encephalopathy (Brandel *et al.*, 2009). Up to 2017, all patients diagnosed with variant Creutzfeldt–Jakob disease had the methionine/methionine (MM) genotype on *PRNP* codon 129. Recently, the first patient with the methionine/valine (MV) genotype on *PRNP* codon 129 with the variant form was reported (Mok *et al.*, 2017).

While the dementia seen in Creutzfeldt–Jakob disease patients is likely a result of prion protein aggregation, various studies suggest that the *PRNP* gene is also involved in other forms of dementia, but results are inconsistent (Rujescu *et al.*, 2003; Del Bo *et al.*, 2006; Jeong *et al.*, 2007; Poleggi *et al.*, 2008; Choi *et al.*, 2010; Golanska *et al.*, 2013; He et al., 2013; Zhang *et al.*, 2016). Furthermore, studies performed on early cognitive decline and early-ons*et Al*zheimer’s disease also show inconsistency regarding which of the homozygous carriers have a higher risk (Croes *et al.*, 2003; Dermaut *et al.*, 2003). In contrast, a meta-analysis on the role of the M129V polymorphism in Alzheimer’s disease suggests that individuals with at least one valine allele have a lower risk of Alzheimer’s disease (He *et al.*, 2013), while the International Genetics of Alzheimer Disease Project did not find an association (Kunkle *et al.*, 2019).

In this study, we report on the association between the *PRNP* M129V polymorphism and mild cognitive impairment and dementia, including Alzheimer’s disease, within the population-based Rotterdam Study.

## Materials and methods

### Setting and study population

The Rotterdam Study is a prospective population-based middle-aged and elderly cohort that started in 1990 in the district of Ommoord, in Rotterdam, The Netherlands. The study includes 14 926 participants and has three subcohorts (Ikram *et al.*, 2017). At start of the study, all inhabitants of the district of Ommoord who were aged 55 years and older were invited to participate. At baseline, in 1990–93, of the 10 215 invited inhabitants, 7983 agreed to participate in the baseline examination (response rate 78%). In 2000, the cohort was extended with 3011 participants (67% of invitees). This extension consisted of all persons living in the study district who had become 55 years and older or had moved into the study district. A second extension was initiated in 2006, in which 3932 participants (65% of invitees) who were 45 years and older were included. Study rounds consist of a home interview and visits with extensive investigations at the dedicated research centre. Rounds are repeated every 4–6 years. Between 2002 and 2005 extensive neuropsychological tests were implemented in the Rotterdam Study (de Bruijn *et al.*, 2014). These tests are a requirement for the assessment of mild cognitive impairment. Participants without contraindications are also invited to undergo a brain MRI scan, which has been implemented routinely since 2005, as discussed previously (Ikram *et al.*, 2015). Participants are continuously monitored for diseases and mortality through linkage of the medical records from the general practitioners and municipality records. The Rotterdam Study has been approved by the Medical Ethics Committee of the Erasmus MC and by the Ministry of Health, Welfare and Sport of The Netherlands. All participants provided written informed consent to participate in the study and to obtain information from their treating physicians.

### Genotyping

A total of 11 496 participants who were genotyped passed genotyping quality control (92% of all subjects with genotyping) (Niemeijer *et al.*, 2015). Exclusion criteria were a call rate <98%, Hardy–Weinberg *P*-value <10^−6^, minor allele frequency <0.01%, excess autosomal heterozygosity >0.336, sex mismatch and outlying identity-by-state clustering estimates. Imputations were performed using the Haplotype Reference Consortium panel (Loh *et al.*, 2016). We selected one single-nucleotide polymorphism within the *PRNP* gene: M129V. Imputation quality for this single-nucleotide polymorphism was high (*r*^2^ = 0.97). We rounded the imputed single-nucleotide polymorphism dosages to 0 (MM), 1 (MV) and 2 (valine/valine (VV)).

### Assessment of mild cognitive impairment

We defined mild cognitive impairment using the following criteria: (i) presence of subjective cognitive complaints, (ii) presence of objective cognitive impairment and (iii) absence of dementia, as previously described (de Bruijn *et al.*, 2014). The first criterion, presence of subjective cognitive complaints, was evaluated by an interview, which consisted three questions on memory complaints and three questions on impaired daily functioning. If the participant confirmed the presence of one of these six complaints or impairments, subjective cognitive complaints were seen as present. The second criterion, presence of objective cognitive impairment, was assessed using a cognitive test battery comprising letter digit test, Stroop test, Purdue Pegboard test, fluency task and 15-word verbal learning test based on Rey’s recall of words. Participants were classified as objectively cognitive impaired if they scored <1.5 standard deviation of the age-adjusted and education-adjusted mean of the study population. This has previously been explained in more detail (de Bruijn *et al.*, 2014). For this study, we included participants with genetic data available and mild cognitive impairment assessment during the implementation period of extensive neuropsychological tests, as a large number of participants were then screened for mild cognitive impairment for the first time.

### Assessment of dementia

Dementia ascertainment involved cognitive screening at the study research centre. We further assessed individuals with a Mini-Mental State Examination score of <26 or a Geriatric Mental State Schedule organic level of >0 (de Bruijn *et al.*, 2015), by administering the Cambridge Mental Disorders of the Elderly Examination by a research physician. We also interviewed spouses or informants. A consensus panel headed by a consultant neurologist established the final diagnosis according to the standard criteria. We studied the outcomes of all-cause dementia (DSM-III-R) and Alzheimer’s disease (NINCDS–ADRDA). For the assessment of dementia, and type of dementia, the latest follow-up information with available data was used to determine the disease state. Follow-up for dementia was near complete until 1 January 2015. Within this period, participants were censored at the date of dementia diagnosis, death or loss to follow-up.

### Brain MRI measurements

Brain MRI scanning was performed using a 1.5-T scanner (General Electric Healthcare, Milwaukee, WI, USA), and it included T_1_-weighted sequence, proton-density-weighted sequence, fluid-attenuated inversion recovery-weighted sequence and T_2_-weighted sequences (Ikram *et al.*, 2015). Details on methods for the measurement of brain volume and hippocampal volume have been described earlier (Ikram *et al.*, 2015).

### Statistical analysis

Logistic regression analyses were used to study the association between the M129V polymorphism and mild cognitive impairment. For mild cognitive impairment as outcome of interest, analyses were conducted in all participants who had mild cognitive impairment assessment at the implementation of neuropsychological tests. Subsequently, the association between the M129V polymorphism and incident dementia, including incident Alzheimer’s disease, was studied using Cox proportional hazard models. For these two outcomes of interest, analyses were conducted in all participants who had dementia status and dementia type assessed. We ran the analyses unadjusted (Model 1) and also while adjusting for age and sex (Model 2). As secondary analyses, we ran linear regression analyses to study the association between the M129V polymorphism and MRI-derived brain volume and hippocampal volume, while adjusting for age, sex and intracranial volume.

### Data availability statement

Because of restrictions based on privacy regulations and informed consent of the participants, data cannot be made freely available in a public repository. Data can be obtained upon reasonable request. Requests for the Rotterdam Study data should be directed towards the management team (secretariat.epi@erasmusmc.nl).

## Results

### Descriptive statistics

**Figure 1:**
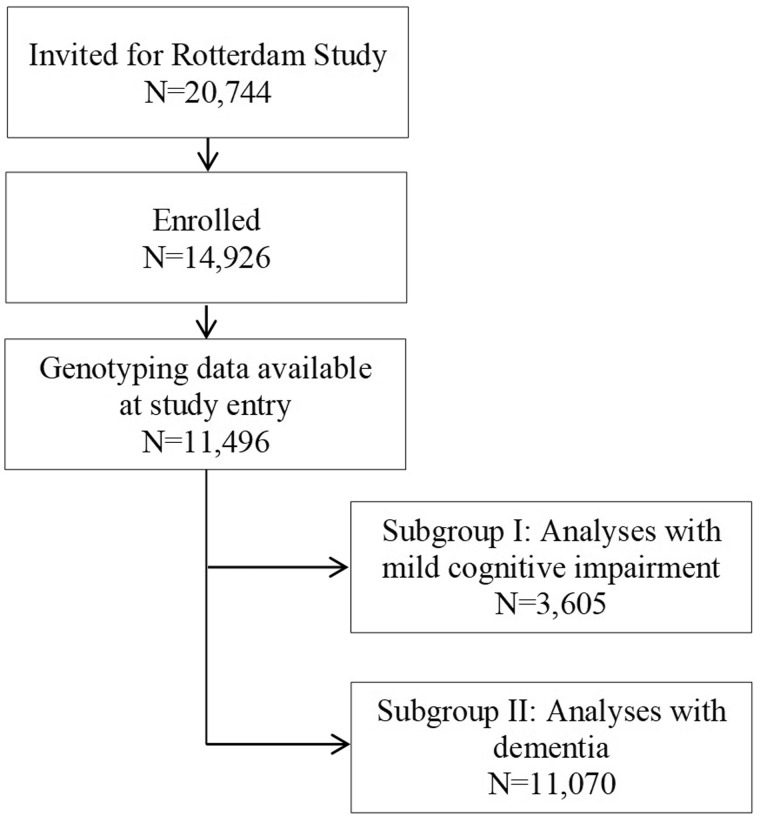
**Selection of subjects from the Rotterdam Study population.** Flowchart of the selection of the participants from the Rotterdam Study.

We examined the 11 496 participants in the Rotterdam Study who had genotyping data available from the M129V polymorphism from the *PRNP* gene (Fig. 1). Of these participants, 4991 (43.4%) had the MM genotype, 5209 (45.3%) had the MV genotype and the remaining 1295 (11.3%) had the VV genotype. The carriers of the MM genotype tended to be slightly older than the two other genotype groups. In the overall population, the distribution of this genotype was in Hardy–Weinberg equilibrium. The general characteristics of the study population are shown in Table 1.

**Table 1:** Characteristics of the study population

	Genotype at codon 129 of the prion protein gene			*P*
	MM	MV	VV	
	*N* = 4991	*N* = 5209	*N* = 1296	
Age at baseline, median (range)	63.2 (45.6–99.1)	62.6 (45.5–99.2)	62.6 (45.7–99.2)	0.06
Sex, female, *n* (%)	2878 (58)	3068 (59)	726 (56)	0.13
*APOE*ε4 carrier, *n* (%)^a^	1377 (29)	1451 (29)	369 (30)	0.82

a Four percent of missings in each genotype group.

*APOE*ε4, apolipoprotein E epsilon 4; *n*, number of people; *N*, number of people at risk; M, methionine; V, valine.

### *PRNP* M129V and the prevalence of mild cognitive impairment

We found that carriers of the MM genotype have a higher prevalence of mild cognitive impairment than those who are heterozygous (odds ratio, 1.40; 95% confidence interval, 1.11–1.78; *P *=* *0.005), as shown in Table 2. Carriers of the VV genotype showed a borderline significant higher prevalence of mild cognitive impairment than heterozygous carriers (odds ratio, 1.37; 95% confidence interval, 0.96–1.97; *P *=* *0.08). Combining homozygous carriers (MM and VV) resulted in an odds ratio of 1.40 (95% confidence interval, 1.11–1.75; *P *=* *0.004). The allele and genotype distribution of the M129V polymorphism was not in Hardy–Weinberg equilibrium in mild cognitive impairment cases. There was an excess of homozygotes (both MM and VV carriers) in the mild cognitive impairment cases.

**Table 2:** Association of *PRNP* M129V polymorphism with mild cognitive impairment

Genotype	Cases, *n* (%)	Controls, *n* (%)	Model 1 OR (95% CI)	*P*	Model 2 OR (95% CI)	*P*
MM	182 (51)	1414 (44)	1.42 (1.13–1.80)	0.003	1.40 (1.11–1.78)	0.005
MV	133 (37)	1471 (45)	1.00 (reference)		1.00 (reference)	
VV	45 (13)	360 (11)	1.38 (0.97–1.98)	0.08	1.37 (0.96–1.97)	0.08

Hardy–Weinberg equilibrium *P*: overall: 0.95, cases: 0.0099, controls: 0.44.

CI, confidence interval; MM, methionine/methionine; MV, methionine/valine; *n*, number of people; OR, odds ratio; VV, valine/valine.

### *PRNP* M129V and other outcomes

We did not find significant evidence for the association of *PRNP* M129V polymorphism with incident dementia, including Alzheimer’s disease, as shown in Table 3. In dementia and Alzheimer’s disease patients and controls, the M129V polymorphism was in Hardy–Weinberg equilibrium. We also did not find a significant association of the M129V polymorphism with brain volume and hippocampal volume.

**Table 3:** Risk of incident dementia, including Alzheimer’s disease, stratified on *PRNP* M129V polymorphism

Genotype	Cases, *n* (%)	Cohort at risk, *n* (%)	Model 1 HR (95% CI)	*P*	Model 2 HR (95% CI)	*P*
Dementia						
MM	602 (43)	4802 (43)	1.01 (0.90–1.13)	0.89	1.01 (0.90–1.13)	0.90
MV	631 (44)	5015 (45)	1.00 (reference)		1.00 (reference)	
VV	176 (13)	1253 (11)	1.12 (0.95–1.32)	0.18	1.16 (0.98–1.37)	0.09
Alzheimer’s disease						
MM	480 (43)	4680 (44)	1.01 (0.89–1.15)	0.84	1.01 (0.87–1.15)	0.82
MV	500 (45)	4884 (45)	1.00 (reference)		1.00 (reference)	
VV	125 (11)	1202 (11)	1.02 (0.84–1.24)	0.87	1.06 (0.87–1.29)	0.56

Hardy–Weinberg equilibrium *P* for dementia: overall: 0.30, cases: 0.59, controls: 0.19. Hardy–Weinberg equilibrium *P* for Alzheimer’s disease: overall: 0.18, cases: 0.76, controls: 0.19.

CI, confidence interval; HR, hazard ratio; MM, methionine/methionine; MV, methionine/valine; *n*, number of people; VV, valine/valine.

## Discussion

In this study, we investigated the association of the M129V polymorphism of the *PRNP* gene with mild cognitive impairment and dementia. We found that homozygous carriers had more often mild cognitive impairment than heterozygous carriers. We did not find an association between this polymorphism and dementia. We found a significantly higher prevalence of mild cognitive impairment in carriers of the MM genotype than in carriers of the MV genotype and a non-significant higher prevalence of mild cognitive impairment in carriers of the VV genotype than in carriers of the MV genotype. In contrast, our results do not indicate a role of the M129V polymorphism of the *PRNP* gene in dementia, including Alzheimer’s disease, nor in brain volume and hippocampal volume. The mechanism underlying the association between the M129V polymorphism of the *PRNP* gene and neurodegeneration is thought to be caused by the effect the M129V polymorphism has on the aggregation of pathological prion proteins in the brain. The aggregation of pathological prion proteins is a biological process similar to the aggregation of tau protein and amyloid accumulation (Walker, 2018), which are key features in Alzheimer’s disease (Bloom, 2014).While most neurodegenerative diseases are known to involve more than one misfolded or abnormally aggregated protein, a recent study demonstrated prion protein aggregation to be an independent pathogenic mechanism, with no cross-seeding between prion protein and misfolded amyloid beta (Rossi *et al.*, 2019), although prion protein is a stress protein that is elevated in (amyloid beta) plaques (Kellett and Hooper, 2009). This suggests that the neurodegenerative changes due to prion protein accumulation could occur regardless of other ongoing neurodegenerative processes. This could be a possible explanation for our finding that the M129V polymorphism of the *PRNP* gene plays a role in mild cognitive impairment, and not in dementia. Our study showed that the M129V polymorphism of the *PRNP* gene may increase the prevalence of mild cognitive impairment. Mild cognitive impairment is often seen as pre-stage of Alzheimer’s disease, related to apolipoprotein E. This study showed the heterogeneity of mild cognitive impairment as also other genes and other non-Alzheimer’s disease dementing disorders are included in the mild cognitive impairment population.

Several limitations in this study should be addressed. Our analyses of the association of the M129V polymorphism with mild cognitive impairment have been performed cross-sectionally. We were not able to calculate the life-time risk of the M129V polymorphism on mild cognitive impairment in the participants from our population-based cohort study. Another potential limitation is the generalizability of our findings, as our study was performed in mainly Caucasians. Previous studies have studied the effect of the M129V polymorphism of the *PRNP* gene in mild cognitive impairment and different types of dementia in Asians (Jeong *et al.*, 2007; Choi *et al.*, 2010; Zhang *et al.*, 2016). No association was found between the M129V polymorphism and mild cognitive impairment in a Korean population (Choi *et al.*, 2010). Our findings are therefore not generalizable to other ethnicities.

Our study also had several strengths. We used a population-based cohort study, and our study had a relatively large sample size. Although the sample size of the participants who underwent initial mild cognitive impairment assessment was smaller than the overall sample with available genotyping data and dementia assessment, we had sufficient power to demonstrate the association between the M129V polymorphism and mild cognitive impairment.

In conclusion, we found a statistically significant higher prevalence of mild cognitive impairment in carriers of the MM genotype in the M129V polymorphism of the *PRNP* gene in the Rotterdam Study, but no associations were found between this polymorphism and incidence of dementia, including Alzheimer’s disease. Future studies should further elucidate the role of the M129V polymorphism of the *PRNP* gene in cognitive function, dementia and other neurodegenerative traits.
